# 

**Published:** 1900-01-15

**Authors:** 


### SUBJECT INDEX TO VOLUME LIV.

Address, President's, Ohio Dental Society, Bethel, 38.
Address, President's, Michigan Dental Association, Raymond, 329.
Address of Welcome to Michigan Dental Association, Westbrook, 325.
Address, in reply to Westbrook, Metcalf, 326.
Address, President's, Southwestern Michigan Society, Runyan, 595.
Address, President's, Northern Indiana Dental Society, Goodwin, 598.
Alternating Current Engine, 520.
Aluminum Solder, Cook, 77.
Alveolar Hemorrhage, Turner, 295.
Amalgams, Flagg's, 203; Pruyn, 207; McClelland, 281; Hunter, 285; Smith, 256; Cassidy, 287; Lang, 287; Heise, 288; Kumler, 288.
Amalgam Crown, Barnes, 523.
Ancient Japanese Dentistry, Long, 42.
Anesthetics, Local, Mann, 509.
Another Century, Diack, 417, 450.
Antiseptic Surgery of the Mouth and Face, Logan, 617.
Antrum Diseases, Oakman, 256, 271, 274, 454; Rix, 272; Watling, 273; Van Doorn, 72; Corns, 451.
Arseneous Acid for Devitalizing Pulps, Beard, 184; Fletcher, 190; Hunter, 190; Smith, 191, 193; Kumler, 191; Locke, 192; Rauh, 192; Rose, 192; Stern, 192; Sage, 193; Callaham, 193; Heise, 195.
Babbit Metal, Hall, 207.
Bacteriology of the Mouth, Ivins, 113; Hunter, 116; Kumler, 117; Heise, 117, 120, 122; Sage, 118; Molyneaux, 120; Rose, 121.
Banquet at Decatur, Michigan, 275.
Bridge-work, Baldwin, 513; Young, 239, 255; White, 252; Rix, 253; Essig, 243; Siddall, 253; Hoff, 255.
Broaches, disinfection of, Hartzel, 317.
Caries, preventing, Williams, 156.
Cataphoresis, disastrous results from using, Bogue, 296.
Cause and Effect, Harvey, 609.
Cavity Preparation, Harper, 402, 406, 412; Siddall, 409; Root, 410; Sweetnam, 410; Crouse, 410; Dorrance, 411, Hoff, 411; Wood, 412.
Children's Teeth, Essig, 217; Hoff, 218, Harper, 219, 222; Siddall, 220; White, 220; Roe, 221; Rix, 221; Fowler, 221; Honey, 221; Runyan, 222; Watling, 223.
Chloral Camphor, Harlan, 522.
Chloretone, Leo, 309.
Clamps, new form, Harvey, 143; Butler, 146; Ambler, 146; Barnes, 147; Jackman, 147; Stephan, 147; Fletcher, 148.
Cleansing the Teeth, Rose, 541.
Cocain and its Antidote, Curtis, 487; Sterilizing its solutions, 313; New method of applying, 316; With silver nitrate, 375.
Compressed Air, Brown, 80.
Congress, Paris International, 525.
Continuous Gum, Jackman, 77.
Contour Filling, Owens, 76.
Dentally Educating the Public, Porter, 338, 350; Hoff, 343; Hall, 346; Root, 347; Metcalf, 347; Crouse, 347.
Dental Register, Incidents in Its History, Hoff, 32, 51, 53, 148. 318.
Dental Protective Association, resolutions, 455.
Dental Wounds, Ottolengui, 204.
Dentin, treatment of softened,

Williams, 154. (See also Sensitive Dentin.)
Dentists as Book-keepers and Collectors, Zinn, 602.
Essential Oils, Prinz, 433.
Eucain, bad result from injection, Overholser, 314.
Extraction, its technique, Guy, 301; Amoore, 307.
Extracting for regulating, Rauh, 289; Callahan, 292; Locke, 293; Williams, 294; Smith, 294; Wright, 295; Hunter, 295.
Fetor from the dental standpoint, B. H. Smith, 466, 480; Brubaker, 476; Schamberg. 477; Thompson, 477; Gaskill, 478; Cupid, 478; Truman, 478: Gaylord, 479; Kirk, 480.
Filling with Automatic Plugger, McLean, 81.
Forced Respiration, Barnes, 79.
Foreign Relations Committee Report, Barrett, 457.
Fusing Metals in Tin, 105.
Future Dentists, Fowler, 413, 450; Blackmarr, 423; Hoff, 425; Loeffler, 428; Root, 429; Woolsey, 429.
Gold Crowns, removal of, Ottolengui, 482.
Gold Crowns, with porcelain face, Evans, 197; Northrop, 198; Littig, 199.
Gold as a filling material, Hoff, 350, 371; Watling, 363; Warboys, 367; Hall, 368; Crouse, 368; Ricks, 371.
Gold Filling in Porcelain Teeth, Weaver, 78.
Gold Wire for Open Joints, Ruggles, 82.
Gum Incising Instruments, Bell, 77.
Gutta-percha Filling, 106, 561.
Haskell's visit to a Liverpool dental office, 586.
Hand Pressure Filling, Scheble, 74.
Hemorrhage at apex of root, Crawford, 316; After extraction, 156.
Hydronaphthol, Monroe, 204.
Impression taking, Bryan, 82; Use milk mouth wash before taking, 375.
Inlays, Van Woert, 200; Evans, 202. (See also Porcelain Inlays.)
Laboratory, dental, Nyman, 205.
Lead, to clean, Mariotte, 524.
Leucokaratosis Buccalis, Leahy, 442, 449; Stern, 447; Hunter, 447; Smith, 447; Cassidy, 448; Heise, 449.
Ludwig's Angina, from abscess, Kennedy, 517.
Malposed Tooth, Peck, 70.
Mason Crown and Bridge, Hulick, 78.
Maxillary Sinuses, empremia of, Custer, 129, 139, 142; Taft, 139; Smith, 139; Fletcher, 140.
Michigan State Dental Association, 325.
Moderation in Office Equipment, 524.
Mummification of Pulps, Flagg, 1; Herbst, 2; Witzel, 3; Baume, 4; Miller, 4; Soderberg, 6, 523; Waas, 11; Boennecker, 14; Houghton, 19; Barker, 25; Brockway, 25; Turner, 26; Campbell, 26; Rippier, 27; Hamlet, 27; Kirk, 27; Prinz, 628.
Nirvanin, 315.
Nitrous Oxid, recent death from, 623.
Ohio State Dental Society, minutes of 1899 meeting, 156.
Old and New in Dentistry, Rowley, 230, 236; Rix, 235, 237; Siddall, 235; Ellsworth, 236; Hoff, 236; Watling, 238; Runyan, 238.
Orthodontia Impressions, Barnes, 80.
Oxyphosphates, Ames, 223; Codding, 227; Rowley, 228; White, 228; Funk, 228; Hoff, 229; Influence on Tooth Tissues, Hoff, 163; Niles, 167; Miller, 167; Burchard, 168; Kirk, 171; Taft, 173; Hunt, 174; Black, 176; Hart, 176; Junkerman, 176; Morgan, 178;

Dunbar, 178; Wedelstaedt, 179; Ames, 180.
Paris International Dental Congress, 525.
Perspiring Hands, 106.
Polishing Filling, 209.
Porcelain in Dentistry, Capon, 549, 576.
Porcelain Inlays, Johnson, 578; Head, 562, 569; Hart, 567; Tracy, 567; Hofheinz, 570; Ottolengui, 571; Rhein, 572; Van Woert, 574; Butler, 576.
Pressure Anesthesia, Cook, 73; Arnold, 81; Higgins, 393, 401; Lee, 394; Stofflet, 396; Essig, 396; Hines, 396; Siddall, 397; Oakman, 398, 400; Root, 399; Corns, 399; Sweetnam, 399; Blackmarr, 401.
Proceedings Southwestern Michigan Dental Society, 217.
Pulp Extirpation Methods, Locherty, 492, 506; Briggs, 500; Gillett, 502; Babcock, 503; Downes, 504; Kimball, 504; Luckey, 505; Leroy, 505; Smith, 506; Bogue, 507; Zerfing, 508.
Pyrozone, a styptic, Kauffer, 264; West, 156; as a bleacher, Stern, 82.
Refining Gold, Kumler, 83.
Repairing Instruments, Lodge, 83.
Replantation, Van Metre, 109; Fletcher, 111; Cassidy, 111; Smith, 111; Molyneaux, 112.
Resolutions on the death of Theo. Menges, 539.
Resolutions on the death of Geo. H. Cushing, 538.
Roentgen Rays, Price, 84, 102; Custer, 97; Cassidy, 99; Taft, 101; Emminger, 101; Blandy, 153.
Root Canals, technique of opening, Hall, 373, 379, 390; Runyan, 386; Wood, 388; Crouse, 388, 389, 392; Oakman, 388; Hoff, 389; Diack, 390; Root, 390
Saliva, alkaline, Fletcher, 122.
Saws for the Dental Engine, Schuller, 456.
Sensitive Dentin, Junkerman, 49, 55, 68; Whinnery, 58; Smith, 60, 63; Custer, 61; Arnold, 62; McLean, 63; Fletcher, 64; Cassidy, 66; Taft, 66; Bogue, 208.
Solder, platinum, McCandless, 208.
Spectacles for Dental Work, Booth, 155.
Teeth Erupted in the Antrum, Fletcher, 70.
Temperaments, Gustavus North, 600.
Tin and Gold Filling, Keeley, 75; Ambler, 76; Butler, 81.
Toothache, mountain, Hofner, 207.
Varnish, removal of excess, 106.
Vulcanite, removal from teeth, 106; finishing, 208; removing teeth from, 156.
Wire Drawing Machine, Stephan, 82; Locke, 83.

EDITORIAL.

Banquets to Drs. Kingsley and McKellops, 265.
Dental Education of the Genius, 431.
Dental Laboratories, 536.
Dental Register's Change of Management, 51.
Dentistry a Specialty of Medicine, 588.
Diagnosis, new aids to, 107.
Dignified Journalism, 482.
Diplomacy and Dentistry, 210.
Doctor of Stomatology Degree, 590.
Doctors Theo. Menges and Geo. H. Cushing, 317.
Donaldson Broach Case, 209.
Indiana Dental Journal, 266.
National Association of Dental Faculties, 430.
National Dental Association, 159, 430.
National Legislation, 159.
National School of Technics, 54.
New Colleges, effort to control organization of, 592.
Ohio State Dental Society, 593.
Operative Clinics at the National Meetings, 265.
Ottolengui's Report of the National Meetings, 431.

Questional Method of Preparing Students for College, 535.
Reply to Editor of *Dental Brief*, 633.
Shall Dentists be Medically Educated? 376.
Smale and Williams, of London, England, 212.
Tardy Appearance, 53.
Testifying in Court, 209.

BIBLIOGRAPHY.

Artificial Crown and Bridge-work, Evans, 639.
Discovery of Anesthesia, Wells, 640
Facts, Fads and Fancies about the Teeth, Ambler, 485.
Malocclusion of the Teeth, Angle, 637.
The Practice Builder, Hambly, 266.

ANNOUNCEMENTS.

American Medical Association, 215.
Detroit Dental Society, 593.
Illinois Board of Examiners, 214.
Illinois State Dental Association, 214.
Indiana, Illinois and Kentucky Tri-State Meeting, 212.
International Dental Congress, transportation, 161.
Kentucky State Dental Association, 213.
Michigan Board of Examiners, 213, 432.
Michigan Dental Association, 161.
Missouri Dental Association, 212.
National Association of Dental Faculties, 212.
National Dental Association, 213.
New York State Dental Association, 214.
Northern Ohio Dental Association, 161, 212.
Officers elected by National Societies, 432.
Ohio State Board of Dental Examiners, 160, 540.
Ohio State Dental Society, 540.
Pennsylvania Dental Examiners, 160.
Pennsylvania State Dental Society, 269.
Section on Etiology and Physiology, 269.
Section on Operative Dentistry, 269.
Southwestern Michigan Dental Society, 594.
The Institute of Dental Pedagogics, 635.
West Virginia State Dental Association, 594.

OBITUARIES.

Burchard, Dr. H. H., 484.
Clowes, Dr. J. W., 537.
Cushing, Dr. Geo. H., 323.
Menges, Dr. Theodore, 319.
Welch, Dr. Charles, 587.

